# Performances of four Nucleic Acid Amplification Tests for the identification of SARS-CoV-2 in Ethiopia

**DOI:** 10.1038/s41598-022-24411-2

**Published:** 2022-11-24

**Authors:** Belete Woldesemayat Hailemariam, Kidist Zealiyas, Gadissa Gutema, Gebremedhin Gebremicael, Sisay Adane, Sisay Tadele, Adamu Tayachew, Shambel Araya, Kassu Desta

**Affiliations:** 1 HIV/AIDS Disease Research Team, TB and HIV/AIDS Disease Research Directorate, Ethiopian Public Health Institute, Addis Ababa, Ethiopia; 2 National Influenza Reference Laboratory, Ethiopian Public Health Institute, Addis Ababa, Ethiopia; 3 Department of Medical Laboratory Sciences, College of Health Sciences, Addis Ababa University, Addis Ababa, Ethiopia

**Keywords:** Biological techniques, Microbiology, Molecular biology, Diseases, Health care, Medical research, Molecular medicine

## Abstract

Since Coronavirus Disease-2019 (COVID-19) outbreak was reported, many commercial Nucleic Acid Amplification Tests (NAAT) have been developed all over the world, and it has been the standard method. Even though several assays were rapidly developed and applied to laboratory diagnostic testing, the performance of these assays was not evaluated in different contexts. Thus, this study aimed to assess the performance of Abbott SARS-CoV-2, Daan Gene, BGI and Sansure Biotech assays by using Composite Reference Standard (CRS). The study was conducted at the Ethiopian Public Health Institute (EPHI) from December 1 to 30/2020. Of the 164 nasopharyngeal samples were extracted by using a QIAamp RNA mini kit and Abbott DNA sample preparation system. Out of 164 samples, 59.1% were positive and 40.9% were negative by CRS. Sansure Biotech positivity was significantly low compared to CRS (p < 0.05). The overall agreement of the four assays compared to CRS was 96.3–100%. The performance of the four assays had almost comparable diagnostic performance, except for a low positive rate of Sansure Biotech assay. Hence, Sansure Biotech assay [Research Use Only (RUO)] needs further verification on its use in Ethiopia. Finally an additional study should be considered for evaluating assays with respective manufacturer claims.

## Introduction

Laboratory testing is an integral part of the World Health Organization (WHO) Strategic Preparedness and Response Plan (SPRP) for Coronavirus Disease-2019 (COVID-19). WHO recommends that countries need to increase the laboratory capacity to boost their level of preparedness, proper case management, alertness and quick response for public health. This indicates that the role of the laboratory is a key to defining the disease characteristics, and epidemiology of an emerging infectious pathogen and in controlling its spread^1^.

COVID-19 diagnosis requires both epidemiological, and medical history information and personal symptoms/signs, as well as radiological and laboratory data^2^. Since the COVID-19 outbreak reported from Wuhan, China, many commercial Nucleic Acid Amplification Test (NAAT) assays have been developed all over the world. And Reverse Transcription real-time Polymerase Chain Reaction (rRT-PCR) has been continued as the routine and standard method for the laboratory diagnosis of Severe Acute Respiratory Syndrome-2 (SARS-CoV-2) infection^3^. Molecular detection of SARS-CoV-2 commonly depends on N (nucleocapsid protein gene), E (envelope protein gene), and RdRp gene (RNA-dependent RNA polymerase gene) in ORF1a/b (Open Reading Frame 1a/b) region identification from the viral genome. These are considered the main conserved regions found in the viral genome for the identification of the virus^4^. Among these genes, the RdRp and E genes had high analytical sensitivity for detection, whereas, the N gene provided poorer analytical sensitivity^5^.

The performance of PCR testing can vary due to various factors such as; the quality of extraction reagent, amplification/detection reagent, method of extraction, PCR machine, and other instrumentations. As of April 2020, more than 48 different diagnostic devices from nine countries had received Emergency Use Authorization (EUA) for the diagnosis of COVID-19^6^. In Ethiopia, more than fourteen types of real-time PCR platforms in 26 governmental public health testing facilities, including the ABI 7500, Abbott m2000, Roche 48000 and Quant-studio are in use for SARS-CoV-2 PCR testing^7^. Similarly, different PCR detection kits are available, such as; Daan Gene assay, Abbott SARS-CoV-2 assay, Sansure Biotech assay and BGI SARS-CoV-2 assay. Although rRT-PCR assay is a highly sensitive technique, false-negative results have still been reported in some COVID-19 patients due to insufficient viral Ribonucleic Acid (RNA) copies in the specimen resulting from the improper collection, transportation, storage, and handling, as well as laboratory testing conditions and personnel operation^8^. Moreover, improper samples or control handling, Cycle threshold (Ct) cutoff value set, and cross-reactions with other pathogens nucleic acids or inactive/residual RNA of SARS-CoV-2 could lead to false positive results in rRT-PCR detection^9^. As a result, it is evident that PCR tests do recognize gene fragment carriers as they do not even distinguish the real active viral genes, and thus tests recognize carriers and not patients^10^. Therefore, diagnostic performance evaluations are very crucial by using the standard methods in our settings. Even though many NAAT reagents are available at Ethiopian Public Health Institute (EPHI) and throughout the country, no comparative performance evaluation has been reported to date. Therefore, this study aimed to assess the comparative performance of commercially available rRT-PCR SARS-CoV-2 detection kits using clinical samples.

## Results

### Characteristics of the study participants

A total of 164 COVID-19 suspected participants were included in this study. The majority of samples were taken from treatment centers (118/164 = 72%) and the remaining 46 (28%) participants were from non-treatment centers. Out of non-treatment center participants, 15 (9.1%) were clinically suspected and 31(18.9%) had contact with confirmed cases. Ninety-three (56.7%) of the participants were male and the mean (± SD) age of the participants was 31.10 (± 11.82) years.

### Performance comparison of SARS-CoV-2 assays

In this study, the rate of positivity and negativity with four COVID-19 testing assays were determined. Hence, the positive rates of Abbott SARS-CoV-2 assay, Daan Gene 2019-nCoV assay, BGI SARS-CoV-2 assay and Sansure Biotech 2019-nCoV assay were 59.1%, 58.5%, 57.9% and 55.5% respectively. The positive and negative rates of Composite Reference Standard (CRS) were 97 (59.1%) and 67 (40.9%) respectively (Table 1). In this study, CRS was defined based on the “any positive” rule, i.e. out of four assay test results, two or more assay test results produced the same results were taken as true positives and negatives.

**Table 1:** Rates of COVID-19 in different SARS-CoV-2 assays.

Types of assays and platforms	Positive (%)	Negative (%)
Abbott SARS-CoV-2 assay	97 (59.1)	67 (40.9)
Daan Gene 2019-nCoV assay	96 (58.5)	68 (41.5)
BGI SARS-CoV-2 assay	95 (57.9)	69 (42.1)
Sansure Biotech 2019-nCoV assay	91 (55.5)	73 (44.5)
Composite reference standard (CRS)	97 (59.1)	67 (40.9)

In this study, we found that the negative percent agreement (NPA) of all assays was 100% (95% CI 94.6–100) compared to the CRS. The lowest PPA was 93.8% (95% CI 87.2–97.1) shown in the Sansure biotech assay and the overall agreement of the Daan Gene 2019-nCoV assay was 99.4% (95% CI 96.6–99.9). In contrast, the overall agreements of the BGI SARS-CoV-2 assay and Sansure Biotech 2019-nCoV assay were 98.8% and 96.3% respectively (Table 2).

**Table 2:** Percent agreement of four SARS-CoV-2 PCR testing assays compared to CRS.

S/n	PCR assay with different PCR platforms	Positive Percent agreement (95% CI)	Negative percent agreement (95% CI)	Overall percent agreement (95% CI)	Cohen’s Kappa value (95% CI)	MacNemar test (p-value)
1	Abbott SARS-CoV-2 assay	100% (96.3–100)	100% (94.6–100)	100% (97.7–100)	1.00	1.00
2	Daan Gene 2019-nCoV assay	99% (94.4–99.8)	100% (94.6–100)	99.4% (96.6–99.9)	0.987 (0.96–1.00)	1.00
3	BGI SARS-CoV-2 assay	97.9% (92.8–99.4)	100% (94.6–100)	98.8% (95.7–99.7)	0.975 (0.927–1.00)	0.50
4	Sansure Biotech 2019-nCoV assay	93.8% (87.2–97.1)	100% (94.6–100)	96.3% (92.2–98.3)	0.925 (0.86–0.975)	0.031*

*Statistically significant difference.

Cohen's Kappa coefficient of agreement between CRS and Abbott SARS-CoV-2 assay result had a perfect agreement (K = 1.00). Similarly, the Cohen’s Kappa value of Daan Gene 2019-nCoV, BGI SARS-CoV-2 and Sansure Biotech 2019-nCoV assays had also perfect agreement with CRS (K ≥ 0.925). In this comparative analysis, the chi-square test (MacNemar test) showed that the result of the Sansure Biotech 2019-nCoV assay was significantly different compared to CRS (p = 0.031) (Table 2).

### Comparative analysis of Ct values in four SARS-CoV-2 PCR assays

**Figure 1:**
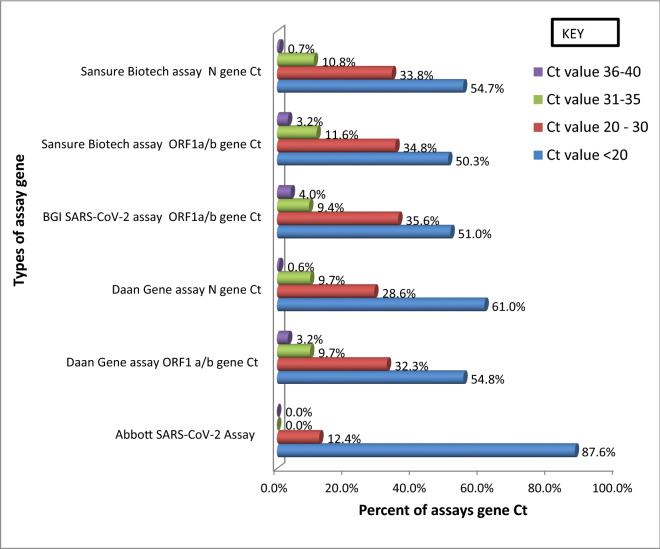
Distribution of Ct value in four assays.

As shown in Fig. 1 the percentage of lowest Ct value (< 20 Ct) of Abbott SARS-CoV-2 assay (combined RdRp and N gene) was 87.6% and ORF1a/b gene Ct value of Sansure Biotech 2019-nCoV assay showed that the percentage of low Ct value (< 20 Ct) was 50.3% and the high Ct value (36–40 Ct) was 3.2%. Ct values greater than 30 were not recorded in the Abbott SARS-CoV-2 assay. On the other hand, on the BGI SARS-CoV-2 assay ORF1a/b gene had a high Ct value (> 36 Ct) percentage was 4% (Fig. 1).

## Discussion

In this study, we compared the diagnostic performance of Abbott SARS-CoV-2 assay (EUA), Daan Gene assay (EUA), BGI SARS-CoV-2 assay (EUA) and Sansure Biotech assay (RUO) with CRS by using 164 nasopharyngeal samples. For all types of assay, RNA isolation and amplification were performed using the recommended methods and kits by their respective manufacturers.

This study showed that the Abbott SARS-CoV-2 assay had equal detection performance with CRS, which had 100% positive, negative and overall agreement. The Cohen’s Kappa agreement was 1.00; this indicated that it has a perfect agreement with CRS. A similar study reported from Washington University, USA showed that the overall sensitivity and specificity of the Abbott SARS-CoV-2 assay were 93% and 100% respectively, compared to CDC-based laboratory- defined assay (LDA)^11^. The Abbott SARS-CoV-2 assay detection system is based on the simultaneous combined detection of N and RdRp genes, since both genes have more sensitivity to minimize false negative results^12^. A study conducted in Vienna, Austria also showed that a high amount of sample volume for extraction and volume of eluate for detection could minimize the dilution effect and increase the detection efficacy^13^. So, the perfect agreement of the Abbott SARS-CoV-2 assay might be due to the detection system of the platform being a simultaneous detection of combined gene, large volume of sample for extraction (0.5 ml) and using a large volume of eluate (40 µl).

Our result also indicated that Daan gene assay detection performance was almost similar compared to the CRS. This is in line with the study conducted at Anhui University, Huainan, China^14^ and with the claim of the manufacturer, which was that the positive coincidence rate was 100%. Even though, concordance result was reported, one sample was falsely negative after retesting on the same eluate, but it was positive in Abbott SARS-CoV-2 and Sansure Biotech nCoV-2019 assays. This indicated that result variability might be seen in different types of assays. Nevertheless, in the study carried out in China^15^, the result of the Daan Gene assay was significantly different (p < 0.05) compared to their lab-defined reference assay. This difference might be due to the sensitivity of the reference assay to detect SARS-CoV-2 and further study might be important to determine the reason.

Additionally, our study also assessed the comparative performance of the BGI SARS-CoV-2 assay with CRS, which showed excellent positive percent agreement (PPA = 97.9%), negative percent agreement (NPA = 100%) and overall percent agreement (OPA = 98.8%). The Cohen’s Kappa value indicated an excellent agreement (K = 0.975). Concordant results have been reported from the studies conducted in the Netherlands^16^ and China^15^. The BGI SARS-CoV-2 assay is a single gene (ORF1a/b) detection assay that uses 10 µl eluate for amplification/detection. Even though, there was an excellent statistical agreement compared with our reference results, the assay missed two positive samples (1.22%) of the total samples. It might have a great clinical impact on the patient and as well as on the transmission dynamics at the community level.

The other comparator assay included in this study was Sansure Biotech nCoV-2019 rRT-PCR assay (RUO); the overall percent agreement was 96.3%. The strength of agreement was also determined by Cohen's Kappa value, which was 0.925, indicating a perfect agreement with CRS. Similarly, our result is the same as the study conducted in Central South University, Changsha district, China^15^, Liuzhou People's Hospital, Department of Clinical Laboratory, Liuzhou, China^17^. Even though the above good statistical concordance was recorded, the chi-square test (MacNemar test) showed that the result of the Sansure Biotech assay has had a statistically significant difference compared to CRS (p < 0.005). Six samples (3.66%) were turned to falsely negative compared with CRS (Supplementary Table 1); this is critical, especially when we consider the virus transmission dynamics. This low detection rate is also supported by the above evidence^15^.

In this study, each detection assay with its respective platform Ct values was determined, and the lowest mean Ct value was recorded on the assay of Abbott SARS-CoV-2. This result might be related to the simultaneous combined gene detection system of the Abbott SARS-CoV-2 assay. Hence, according to Fig. 1, 87.6% of the Abbott SARS-CoV-2 result Ct value was laid on less than 20. Only a few sample results (12.4%) were laid between Ct values of 20–30. There were no Ct values recorded above 30. In addition to Abbott SARS-CoV-2 using the combined gene detection format, this result might be related to the lower limit of detection was comparatively low (32.5 RNA copies/ml)^18^, it was three times lower than the company’s claim of lower limit (100 RNA copies/ml)^19^.

This study has some limitations: First; we did not have a standard/reference method [like; viral load or other Laboratory Defined Assay (LDA)] due to resource limitation. Second: All samples used in this study were nasopharyngeal swab, while the result will not apply for other sample types, and third: our sample size was low.

## Conclusion

This study compared the performance of four SARS-CoV-2 rRT-PCR detection assays by using nasopharyngeal samples. All detection assays except the Sansure Biotech assay have almost comparable performance. Besides, the low positivity rate was identified in the Sansure Biotech assay compared to the CRS (p < 0.05). Sansure Biotech nCoV-2019 assay (RUO) PPA, NPA, and overall agreement was greater than 93.5% and Cohen's Kappa agreement strength value was 0.925. Finally, Sansure Biotech assay (RUO) needs further verification on its use in Ethiopia and an additional study should be considered for the evaluation of respective manufacturer claims.

## Methods

### Study design and setting

A comparative study design was conducted at four health facilities in Addis Ababa, which were Eka kotebe hospital, millennium hall treatment center, Zewuditu memorial hospital and St. Peter TB specialized hospital. Data were collected between December 1 to 31/2020. Health facilities for this study were chosen purposively based on their high number of cases and the major treatment centers found in the city. Similarly, instruments including ABI 7500 and Abbott m2000 real-time PCR instruments were selected based on the NAAT reagent manufacturer’s recommendation and four PCR test kits were selected in this study because of the majority of laboratories found in Ethiopia used at least one of the four (Daan Gene assay, Abbott SARS-CoV-2 assay, Sansure Biotech assay and BGI SARS-CoV-2 assay) assays during the study period.

One hundred sixty-four (164) clinical nasopharyngeal samples were collected by using 3 ml Viral Transport Media (VTM) (Miraclean Technology, Shenzhen, China) from patients who were under investigation for COVID-19 and referred to EPHI for SARS-CoV-2 testing from December 1 to 30/2020. Nasopharyngeal samples were collected by trained sample collectors and transported to EPHI by triple packaging. Each sample was assigned a unique identification number before nucleic acid extraction. The extraction was carried out immediately upon arrival by using both manual and automated extraction methods from each sample. Thus, for Abbott m2000 automated extraction 1.3 ml (including 0.8 ml dead volume and 0.5 ml input volume for extraction) sample was taken from each sample and extracted by Abbott DNA sample preparation system (Abbott Molecular Inc. des Plaines, IL, USA) with a batch of 96 [92 samples, two assay controls and two no-template controls (NTC)] were included throughout the procedure (in extraction and detection) of Abbott Real-time SARS-CoV-2 (EUA) for two rounds. Similarly, for manual extraction, the same samples (which used in automated extraction and detection) were used. Hence, 140 µl samples were aliquoted and extracted by using QIAamp viral RNA mini kit (QIAGEN GmbH, Hilden, Germany) with a batch of 24 (including 20 samples, two assay controls and two NTCs) throughout the procedure for nine rounds. Amplification and detection of manual extracted eluate was performed with BGI SARS-CoV-2 assay, Daan Gene assay and Sansure Biotech assay by ABI 7500 PCR machine.

Automated SARS-CoV-2 viral RNA isolation and purification were performed using Abbott DNA sample preparation reagents by the principle of magnetic beads. Sample inactivation and solubilization of viral particles were done with a detergent, which contains guanidine iso-thiocyanate for protein denaturation and RNase inactivation. Then RNA separated from proteins through solid-phase separation using silica; i.e. nucleic acid bind to silica (SiO_2_) is facilitated by guanidinium salts and the alkaline pH of the lysis buffer. Washing steps could remove any remnant protein and debris to produce a clear solution. The clear RNA separated from silica-based micro particles are by using the magnetic field of the instrument^20,21^. On the other hand, manual RNA extraction and purification were performed via the spin column method and separation of micro-particle from eluate, using centrifugation rather than magnetic rack in the spin column method^21^.

### Abbott real-time SARS-CoV-2 assay (EUA)

The Abbott Real-time SARS-CoV-2 assay (Abbott Molecular, Inc.) test was performed as described in the manufacturer’s instruction, which has received EUA from WHO and FDA^19,22^. In this protocol pre-extraction sample inactivation was performed with a water bath at 56 °C for 30 min^23^, after viral inactivation, nucleic acid extraction was performed from 0.5 ml VTM on the Abbott m2000 SP instrument and using the Abbott m2000 DNA Sample Preparation System according to the manufacturer’s recommendations. The amplification and detection were performed by Abbott m2000 RT-PCR instrument targeted to dual-target assay for the RdRp and N genes. The SARS-CoV-2 and IC-specific probes are each labeled with a different fluorophore, Carboxyfluorescein (FAM), Carboxy-X-rhodamine (ROX), and VIC P (Proprietary dye) for target and internal control detection, thus allowing for simultaneous detection of both amplified products^19^.

### Daan gene nCoV-2019 assay (EUA)

The amplification and detection method of this kit is based on the one-step RT-PCR technique. ORF1a/b and N genes were selected as the conserved region of Daan Gene technology for amplification and detection of target regions. Specific primers and fluorescent probes are designed (the N gene probe is labeled with FAM and ORF1a/b probe with VIC) for detecting SARS-CoV-2 RNA in the specimens. The final eluent and master mix preparation was 5 µl of eluate added to 20 µl of master mix for a final volume of 25 µl. Amplification and detection were performed on ABI 7500 real-time-PCR instrument simultaneously^24^.

### Sansure Biotech detection assay (RUO)

The Sansure Biotech nCoV-2019 nucleic acid diagnostic kit (PCR-florescence probing) was used for detecting ORF1a/b and N genes. The specific probe for each target gene is prepared, the FAM channel is selected for the ORF1a/b region and the ROX channel is for the N gene. In this detection kit, eluate and master mix reagent addition were as follows; 30 µl master mix reagent and 20 µl eluted samples were prepared for detection/amplification. ABI 7500 real-time PCR was used for amplification/detection^25^.

### BGI SARS-CoV-2 testing assay (EUA)

BGI SARS-CoV-2 assay is a real-time fluorescent rRT-PCR Kit for the diagnosis of COVID-19. The target region is found in the ORF1a/b region of the SARS-CoV-2 genome and it is a single-gene based testing assay. Furthermore, the human housekeeping gene β-actin is the target gene for internal control. The master mixing was done by mixing 20 µl master mix reagent and 10 μl of the extracted sample RNA to the well plate^26^. ABI 7500 real time PCR instrument was used for amplification and detection. All nucleic acid amplifications, each assay PCR cycling condition and result interpretation were performed based on respective manufacturer instructions (Table 3).

**Table 3:** Summary of PCR cycling and result interpretation of four assays.

Type of assay	Cycle/s	Temp °C	Duration	Result interpretation
Abbott SARS-CoV-2 assay				The results and interpretations were reported automatically by Abbott m2000 real-time-PCR workstation. If the target and internal control amplification was detected the result was displayed as “positive” and if the target was not amplified and internal control amplification was detected the result displayed as “negative”. Error codes and invalid results also displayed based on the error types^19^
Daan Gene nCoV-2019 assay	1 cycle	50	15 min	The result was interpreted as "positive" if the target gene (N) (labelled with FAM), ORF1a/b (labeled with VIC), and internal control (labelled with Cy5) were detected and Ct values were ≤ 40. If the result had no amplification curve or Ct value > 40 in the FAM and VIC channels and there was an amplification curve in the Cy5 channel, the result interpreted as “negative” If the Ct value of a sample was ≤ 40 in a single channel of FAM or VIC, and no amplification curve was detected in the other channel, interpreted as “retested the sample” and retested result was taken as a final result, if it was positive or negative^24^
	1 cycle	95	15 min	
	45 cycles	94	15 s	
		55	45 s	
BGI SARS-CoV-2 assay	1 cycle	50	20 min	The presence of an amplification curve in the FAM channel (ORF1a/b target region) with a Ct value less than 37 was interpreted as "positive," and the presence of an amplification curve in the VIC channel (internal control) with a Ct value less than 35. The sample was reported as “negative” when, there was no amplification curve in the FAM channel and there was an amplification curve in the VIC channel with a Ct value was less than 35. All samples with their VIC channel amplification curve were not detected or the Ct values greater than 35 were retested and the results of after retesting were taken as the final results^26^
	1 cycle	95	10 min	
	40 cycles	95	15 s	
		60	30 s	
Sansure Biotech nCoV-2019 assay	1 cycle	50	30 min	If there was an amplification curve in the FAM (target ORF1a/b) channel and or an amplification curve was observed in the ROX channel (target N gene) with Ct value was ≤ 40 without consideration of internal control amplification curve (Cy5 channel), interpreted as “positive” If there was no amplification curve in both the FAM and ROX channels and an amplification curve in Cy5 channel was interpreted as “negative”. If there was no amplification curve in all channels or the Ct value was greater than 40 was retested the sample^25^
	1 cycle	95	1 min	
	45 cycles	95	15 s	
		60	30 s	

### Interpretation of the CRS

In this comparative analysis study, we have not used the reference standard method to determine percent agreements (positive, negative and overall) and other comparative parameters of the four assay methods. Each assay comparison was performed against CRS, in this study, CRS was established by “any positive” rule and the result was defined rather than by a single assay test, we used at least two consistent assays test result. Moreover, in the case of COVID-19 transmission scenarios false negative results have a more harmful effect than false-positive results. So, to maximize the accuracy of the CRS result to say “positive” at least two assay test results should have positive, it means at least one of the positive results could be from the EUA assay. Therefore, out of four assay test results, two or more assay test results producing the same results were taken as true positives or negatives^18,27^.

### Data processing and analysis

The data were collected using structured data extraction form, data entry and analysis was done using excel and SPSS version 23.0 statistical software for descriptive statistics. Positive, negative and overall percent agreements were analyzed and Kappa Estimator was employed to determine the strength of agreement of each method with the CRS. The Kappa values were interpreted as follows; from 0.01 to 0.20 slight agreement; from 0.21 to 0.40 fair agreements, 0.41–0.60 moderate agreement, 0.61–0.80 substantial agreement and 0.81–0.99 perfect agreement^28^.

### Ethical considerations

Ethical clearance was obtained from Addis Ababa University and all experimental protocols of the study were approved by the Ethiopian Public Health Institute Scientific ethical review committee. The reference number of EPHI ethical clearance was EPHI/IRB-279-2020. All methods were performed in accordance with the guidelines and regulations of Ethiopian national comprehensive COVID-19 management handbook. Moreover, informed written consent was obtained from all study participants prior to participate in the study.

## Supplementary Information

Supplementary Table 1.

## Data Availability

All data generated or analysed during this study are included in this published article. The data that support the findings of this study are available from the corresponding author on reasonable request.
