# Supplementary material for: Performances of four Nucleic Acid Amplification Tests for the identification of SARS-CoV-2 in Ethiopia
Source: Sci Rep. 2022 Nov 24;12:20282. doi: 10.1038/s41598-022-24411-2 (PMC9700788; doi:10.1038/s41598-022-24411-2)
Supplement: Supplementary file 1 — Supplementary Table 1. [file 41598_2022_24411_MOESM1_ESM.pdf]

Performances of four Nucleic Acid Amplification Tests for the identification of SARS-CoV-2 in Ethiopia

Belete Woldesemayat Hailemariam[1, 3*], Kidist Zealiyas[1], Gadissa Gutema[1], Gebremedihin Gebremicael[1], Sisay Adane[1,], Sisay Tadele[1], Adamu Tayachew[2], Shambel Araya[3], Kassu Desta[3]

1. HIV/AIDS disease research team, TB and HIV/AIDS Disease Research Directorate, Ethiopian Public Health Institute, Addis Ababa, Ethiopia
2. National Influenza reference laboratory, Ethiopian Public Health Institute, Addis Ababa, Ethiopia
3. Department of Medical Laboratory Sciences, College of Health Sciences, Addis Ababa University, Addis Ababa, Ethiopia

Supplementary table 1 (S Table 1): Discordant result in four assays with respective Ct values.

Sample ID	Abbott SARS-CoV-2 assay		Daan Gene 2019-nCoV assay			BGI SARS-CoV-2 assay		Sansure Biotech 2019-nCoV assay		
	Result	Ct value	Result	Ct value		Result	Ct value	Result	Ct value	
				ORF 1a/b	N Gene		ORF 1a/b		ORF 1a/b	N gene
E-012	Positive	16.7	Positive	27.2	26.4	Positive	25.9	Negative	ND	ND
E-032	Positive	15.0	Positive	26.1	24.2	Positive	26.0	Negative	ND	ND
E-054	Positive	11.5	Positive	22.7	21.5	Positive	25.0	Negative	ND	ND
E-123	Positive	18.8	Positive	31.5	30.6	Positive	29.8	Negative	ND	ND
E-125	Positive	12.3	Positive	24.6	21.3	Positive	23.2	Negative	ND	ND
E-150	Positive	27.4	Negative	ND	ND	Negative	ND	Positive	38.7	36.8
E-156	Positive	12.3	Positive	24.6	21.3	Positive	23.2	Negative	ND	ND
E-164	Positive	25.5	positive	36.1	35.8	Negative	ND	positive	36.7	35.4

SARS-CoV-2; Severe Acute Respiratory Syndrome Coronavirus-2, Ct; Cycle Threshold, ORF; Open Reading Frame, nCoV; Novel Coronavirus, ND; Not Detected, RT-PCR; Real-time Polymerase Chain Reaction
